# Burden of Encephalitis in Low- and Middle-Income Countries: 1990–2023

**DOI:** 10.1159/ned/adwag009

**Published:** 2026-08-19

**Authors:** Kun Fang, Huawei Zhang, Jinming Han, Jiahao Song, Xiangjun He, Xingxu Ji, Chengliang Yin, Changqing Liu, Daiquan Gao

**Affiliations:** a Department of Neurology, Xuanwu Hospital, Capital Medical University, Beijing, China; b School of Mental Health, Capital Medical University, Beijing, China; c Department of Neurosurgery, Beijing Chao-Yang Hospital, Capital Medical University, Beijing, China; d School of Basic Medical Sciences, Capital Medical University, Beijing, China; e Department of Biomedical Engineering, Faculty of Engineering, Universiti Malaya, Kuala Lumpur, Malaysia; f Department of Clinical Nutrition, Capital Medical University, Beijing, China

**Keywords:** Encephalitis, Low- and middle-income countries, Age-period-cohort analysis, Joinpoint regression, Epidemiology

## Abstract

**Background:**

Encephalitis remains a major cause of neurological morbidity and mortality worldwide, particularly in resource-limited settings. This study analyzed the long-term trends and disparities in encephalitis burden across 131 low- and middle-income countries (LMICs) from 1990 to 2023.

**Methods:**

Data were obtained from the Global Burden of Disease 2023 study for 131 LMICs. Incidence, prevalence, deaths, and disability-adjusted life years (DALYs) were analyzed using absolute counts and age-standardized rates. Trends were assessed with estimated annual percentage changes, Joinpoint regression, age-period-cohort analysis, correlations with sociodemographic index (SDI), and decomposition analysis.

**Results:**

In 2023, encephalitis in LMICs caused 1.59 million incident cases, 5.21 million prevalent cases, 70,042 deaths, and 4.07 million DALYs. From 1990 to 2023, incident cases increased by 24.7%, while the age-standardized incidence rate fell from 29.57 to 25.15 per 100,000 person-years (EAPC, −0.71; 95% CI, −0.83 to −0.59). Age-standardized mortality and DALY rates also declined, with EAPCs of −1.97 and −2.20. Incidence reached a low point around 2010 before rising again, particularly after 2019. The burden was greatest in children and older adults. Higher SDI was associated with lower age-standardized incidence, mortality, and DALY rates (ρ = −0.59, −0.41, and −0.56, respectively; all *p* < 0.001). Population growth accounted for most of the increase in case numbers.

**Conclusion:**

Encephalitis remains a substantial health burden in LMICs. Although age-standardized mortality and DALY rates have declined, the growing absolute number of cases, recent upward shift in incidence, and persistent concentration of burden among children, older adults, and lower-SDI settings point to continuing gaps in prevention, surveillance, diagnosis, and care.

## Introduction

Encephalitis is a severe inflammatory disorder of the brain parenchyma and an important cause of central nervous system infection-related morbidity and mortality worldwide. Although both infectious and immune-mediated mechanisms are involved, infectious causes account for a large proportion of cases, with viruses being the leading pathogens [1]. Herpes simplex virus is a major sporadic cause globally, whereas Japanese encephalitis virus and other arboviruses continue to contribute substantially in endemic and resource-limited settings. Because encephalitis encompasses infectious, post-infectious, and immune-mediated conditions, its epidemiological patterns may vary according to etiology, diagnostic capacity, and access to treatment. Owing to its high risk of death and persistent neurological sequelae, encephalitis remains an important clinical and public health challenge across both pediatric and adult populations [2–5].

Encephalitis remains a substantial global health burden, with around 1.5 million incident cases and nearly 92,000 deaths estimated in 2021 [6]. In LMICs, this burden is closely tied to uneven vaccination coverage, fragile surveillance systems, limited pathogen-specific diagnostic capacity, and restricted access to neurological and critical care [7]. These constraints contribute not only to delayed recognition and treatment but also to underestimation of the true burden in routine health systems [7]. While global burden analyses have outlined the overall magnitude and long-term trends of encephalitis, they provide less detail on the epidemiological patterns and health-system challenges most relevant to LMICs [8].

LMICs bear a disproportionate share of encephalitis-related morbidity and mortality [6]. This pattern is particularly evident in South Asia, sub-Saharan Africa, and parts of Latin America, where continued exposure to neurotropic pathogens intersects with vector-borne transmission, gaps in vaccine coverage, limited access to cerebrospinal fluid testing and pathogen-specific diagnostics, and weak surveillance and neurocritical care systems [8–10]. In recent years, these vulnerabilities may have been further amplified by service disruptions during the COVID-19 pandemic, which affected immunization, case detection, and timely access to care. Taken together, these conditions shape not only the occurrence of encephalitis but also the risk of delayed diagnosis, poor outcomes, and under-recognition in routine health systems [8–10]. In this context, encephalitis in LMICs is better understood not only as a neurological syndrome but also as a marker of inequality in infection prevention, diagnostic capacity, and public health preparedness.

More recently, the epidemiology of encephalitis may have been further modified by environmental change, shifts in pathogen ecology, and the direct and indirect effects of the COVID-19 pandemic, particularly in resource-limited settings [8, 11]. However, LMIC-focused evidence on long-term temporal trends, demographic disparities, and development-related gradients remains insufficient. Using data from the Global Burden of Disease (GBD) 2023 study, we quantified the burden of encephalitis in 131 LMICs from 1990 to 2023, including incidence, prevalence, mortality, and disability-adjusted life years. We further examined differences by age, sex, region, income group, and sociodemographic development. By focusing on the settings with the greatest burden, this study aims to provide evidence relevant to surveillance, prevention, diagnostic prioritization, and health-system planning for encephalitis in resource-limited countries.

## Methods

### Data Source and Case Definition

We obtained encephalitis burden estimates from the GBD 2023 study through the Global Health Data Exchange (GHDx) Results Tool [12]. GBD 2023 provides annually updated estimates for 375 diseases and injuries across 204 countries and territories from 1990 to 2023, stratified by age, sex, year, and location. In the GBD framework, incidence and prevalence are estimated using DisMod-MR 2.1, mortality is estimated using the Cause of Death Ensemble model (CODEm), and DALYs are calculated as the sum of years of life lost and years lived with disability [12]. This study followed the Guidelines for Accurate and Transparent Health Estimates Reporting (GATHER) (online suppl. Table 1; for all online suppl. material, see https://doi.org/10.1159/ned/adwag009) [13].

Encephalitis was identified according to the GBD cause definition based on the International Classification of Diseases, 10th Revision (ICD-10: A83-A85.2, A85.8-A86.0, B94.1, F07.1, G04-G05.8, and Z24.1). A list of abbreviations is provided in online supplementary Table 2.

### Definition of LMICs and Sociodemographic Development

LMICs were defined according to the World Bank income classification based on 2023 gross national income (GNI) per capita estimated using the Atlas method [14]. Countries classified as low income (GNI per capita of USD 1,145 or less), lower-middle income (USD 1,146–4,515), or upper-middle income (USD 4,516–14,005) were included in the present analysis, yielding a total of 131 countries and territories. This classification was applied as a static grouping framework across the study period to facilitate cross-country comparability and therefore did not reflect transitions in income category between 1990 and 2023.

Sociodemographic development was assessed using the sociodemographic index (SDI), a composite indicator in the GBD study defined as the geometric mean of lag-distributed income per capita, mean educational attainment among individuals aged 15 years or older, and total fertility rate in women younger than 25 years. Country-specific information on ISO 3 codes, 2023 GNI per capita, income group, SDI values, and SDI groupings is provided in online supplementary Table 3.

### Burden Measures

We assessed encephalitis burden using incidence, prevalence, mortality, and DALYs. For each metric, we extracted absolute numbers and age-standardized rates, including the age-standardized incidence rate (ASIR), age-standardized prevalence rate (ASPR), age-standardized mortality rate (ASMR), and age-standardized DALY rate (ASDR). Age standardization was performed using the GBD global standard population to enable comparisons across countries and over time. All estimates are reported with 95% uncertainty intervals (UIs), defined by the 2.5th and 97.5th percentiles of 1,000 posterior draws generated in the GBD estimation process [12].

### Trend Analysis

Temporal trends in age-standardized rates were quantified using the estimated annual percentage change (EAPC). EAPC was derived by fitting a linear regression model to the natural logarithm of the age-standardized rates over calendar year, expressed as ln⁡(rate) = *α* + *β* × year, where β represents the annual change in the logarithm of the rate. The EAPC was calculated as 100 × (e^*β*^−1), with corresponding 95% confidence intervals (CIs). A positive EAPC indicated an increasing trend, whereas a negative EAPC indicated a decreasing trend over the study period.

To identify potential inflection points in temporal trends, we additionally performed Joinpoint regression for age-standardized rates. The study period from 1990 to 2023 was segmented into multiple linear intervals, and the annual percentage change (APC) for each segment and the average annual percentage change (AAPC) for the full period were estimated [15]. Model selection was based on Monte Carlo permutation testing with 4,499 permutations and Bonferroni adjustment [16]. Joinpoint analyses were applied to the overall LMIC estimates and World Bank income group-specific trends; where applicable, supplementary analyses were also performed for additional burden metrics.

### Age-Period-Cohort Analysis

Age-period-cohort analysis was conducted to examine the independent effects of age, calendar period, and birth cohort on encephalitis incidence, prevalence, mortality, and DALY rates in LMICs. We fitted a log-linear age-period-cohort model in which the logarithm of age-specific rates was expressed as the additive effects of age, period, and cohort. In this framework, age effects reflect biological and clinical vulnerability across the life course, period effects capture temporal influences affecting all age groups simultaneously, and cohort effects represent generational differences related to early-life exposures and long-term contextual conditions [17].

Age-period-cohort results were summarized using the net drift, local drift, longitudinal age curve, period rate ratio, and cohort rate ratio (CRR). Net drift represents the overall annual log-linear change across periods and cohorts, whereas local drift reflects age-specific annual log-linear change over time [18]. Period rate ratios and CRRs were estimated relative to the corresponding reference period and reference cohort after adjustment for age and the nonlinear effects of period and cohort. The longitudinal age curve describes fitted age-specific rates in the reference cohort after adjustment for period effects [19].

### Decomposition and SDI Analyses

To quantify the drivers of changes in absolute encephalitis burden between 1990 and 2023, we performed decomposition analyses for incident cases, prevalent cases, deaths, and DALYs. For each metric, the overall change between 1990 and 2023 was partitioned into contributions from population growth, population aging, and changes in age-specific rates. To assess the association between encephalitis burden and sociodemographic development, we examined correlations between SDI and age-standardized burden metrics in 2023 using Spearman’s rank correlation.

### Statistical Analysis

All analyses were performed using R software (version 4.4.2). All statistical tests were two sided, and a *p* value <0.05 was considered statistically significant where applicable. Because this study was based on modeled GBD estimates, uncertainty was primarily expressed using 95% UIs for burden estimates and 95% CIs for trend statistics derived from regression models.

## Results

### Overall Burden and Temporal Trends of Encephalitis in LMICs

In 2023, LMICs recorded 1,588,958 (95% UI: 1,435,296–1,746,266) incident cases, 70,042 (95% UI: 47,039–109,871) deaths, 5.21 million prevalent cases, and 4.07 million DALYs attributable to encephalitis (Table 1; online suppl. Table 4). From 1990 to 2023, the number of incident cases increased by 24.7%, whereas the ASIR declined from 29.57 to 25.15 per 100,000 person-years (EAPC = −0.71, 95% CI: −0.83 to −0.59) (Table 1). During the same period, prevalent cases also increased in absolute terms, while the ASPR showed an overall downward trend (online suppl. Table 4).

**Table 1.** Incident cases, deaths, ASIRs, and ASMRs of encephalitis in World Bank LMICs, by sex, World Bank income group, and the 20 most populous LMICs, 1990–2023

Characteristics	Incident cases			ASIR			Deaths			ASMR		
	1990 (95% UI)	2023 (95% UI)	percentage change (95% CI), %	1990, per 100 000 (95% UI)	2023, per 100 000 (95% UI)	EAPC (95% CI), %	1990 (95% UI)	2023 (95% UI)	percentage change (95% CI), %	1990, per 100 000 (95% UI)	2023, per 100 000 (95% UI)	EAPC (95% CI), %
Total LMICs	1,273,893 (1,119,425, 1,433,350)	1,588,958 (1,435,296, 1,746,266)	24.73 (21.83, 28.22)	29.57 (23.17, 37.50)	25.15 (20.10, 31.28)	−0.71 (−0.83, −0.59)	74,378 (43,081, 114,594)	70,042 (47,039, 109,871)	−5.83 (−13.34, 1.68)	1.96 (1.04, 3.20)	1.13 (0.70, 1.82)	−1.97 (−2.15, −1.79)
Sex												
Male	656,418 (577,738, 738,372)	821,881 (743,536, 905,073)	25.21 (22.58, 28.70)	30.46 (23.85, 38.61)	25.83 (20.61, 32.15)	−0.71 (−0.81, −0.60)	36,433 (18,477, 61,621)	35,873 (19,847, 63,167)	−1.54 (−6.01, 2.94)	1.97 (0.92, 3.46)	1.18 (0.63, 2.19)	−1.87 (−2.07, −1.67)
Female	617,475 (542,022, 696,557)	767,077 (691,258, 842,634)	24.23 (20.97, 27.53)	28.82 (22.56, 36.52)	24.51 (19.57, 30.52)	−0.73 (−0.86, −0.60)	37,945 (17,327, 69,875)	34,169 (19,817, 60,605)	−9.95 (−23.77, 3.87)	1.98 (0.80, 4.00)	1.08 (0.57, 2.00)	−2.10 (−2.26, −1.94)
World Bank income group												
Low income	50,342 (43,704, 58,187)	96,492 (84,240, 110,776)	91.67 (90.38, 92.75)	15.09 (13.39, 17.08)	13.93 (12.39, 15.64)	−0.24 (−0.26, −0.23)	4,555 (1,972, 8,703)	6,653 (3,448, 12,885)	46.06 (−40.76, 255.72)	1.37 (0.63, 2.50)	1.01 (0.53, 1.95)	−1.07 (−1.16, −0.98)
Lower-middle income	856,662 (762,580, 956,490)	1,145,817 (1,045,769, 1,246,593)	33.75 (30.33, 37.14)	50.15 (45.43, 55.08)	40.23 (37.09, 43.58)	−0.96 (−1.13, −0.80)	50,871 (29,360, 79,438)	51,833 (34,891, 81,729)	1.89 (−41.44, 78.69)	3.77 (2.15, 5.78)	2.10 (1.41, 3.26)	−2.01 (−2.19, −1.83)
Upper-middle income	366,888 (313,140, 418,672)	346,650 (305,288, 388,897)	−5.52 (−7.11, −2.51)	16.58 (14.35, 18.76)	14.09 (12.25, 15.96)	−0.55 (−0.61, −0.50)	18,952 (11,749, 26,453)	11,556 (8,700, 15,257)	−39.02 (−57.57, −12.13)	0.88 (0.56, 1.22)	0.46 (0.33, 0.63)	−2.42 (−2.64, −2.20)
Top 20 populous countries												
India	615,142 (551,229, 678,554)	768,295 (707,870, 836,763)	24.90 (20.09, 31.13)	74.09 (67.62, 80.95)	58.89 (54.25, 63.77)	−1.12 (−1.37, −0.87)	36,134 (21,413, 55,828)	32,615 (22,704, 50,200)	−9.74 (−48.13, 72.25)	5.81 (3.35, 8.78)	2.75 (1.93, 4.24)	−2.61 (−2.86, −2.36)
China	253,116 (215,917, 288,355)	199,725 (173,085, 230,356)	−21.09 (−25.05, −17.02)	22.27 (19.13, 25.37)	17.50 (14.81, 20.44)	−0.33 (−0.47, -0.19)	10,239 (5,491, 16,457)	2,094 (1,439, 3,238)	−79.55 (−88.69, −55.08)	0.93 (0.51, 1.50)	0.19 (0.12, 0.30)	−5.45 (−6.06, −4.85)
Indonesia	18,701 (14,791, 23,207)	25,338 (21,069, 30,077)	35.49 (22.26, 48.67)	9.26 (7.47, 11.20)	10.24 (8.68, 12.03)	−0.60 (−0.89, −0.32)	2,982 (808, 6,281)	2,618 (974, 5,064)	−12.23 (−61.87, 137.45)	1.46 (0.44, 3.05)	1.09 (0.36, 2.17)	−1.06 (−1.19, −0.92)
Nigeria	20,376 (17,687, 23,441)	49,040 (43,030, 55,754)	140.67 (133.21, 148.46)	20.74 (18.43, 23.33)	19.56 (17.60, 21.66)	−0.15 (−0.18, −0.12)	1,960 (840, 4,104)	3,067 (1,472, 6,644)	56.52 (−42.07, 296.97)	1.95 (0.88, 3.74)	1.23 (0.60, 2.55)	−1.71 (−1.91, −1.51)
Pakistan	62,779 (55,782, 70,803)	131,937 (118,855, 145,367)	110.16 (100.91, 119.57)	54.28 (49.26, 59.93)	54.67 (50.04, 59.47)	0.08 (0.05, 0.12)	3,640 (1,593, 7,144)	6,213 (2,767, 12,163)	70.68 (−26.07, 302.12)	4.03 (1.68, 7.79)	3.19 (1.40, 6.36)	−0.51 (−0.60, −0.42)
Brazil	10,692 (9,412, 12,079)	14,138 (12,343, 16,157)	32.23 (22.05, 42.59)	7.10 (6.34, 7.86)	6.88 (5.90, 7.95)	−1.39 (−1.72, −1.04)	509 (444, 574)	670 (584, 765)	31.57 (14.51, 50.36)	0.35 (0.31, 0.40)	0.30 (0.26, 0.35)	−0.73 (−1.07, −0.38)
Bangladesh	62,378 (52,670, 73,077)	75,177 (64,896, 87,299)	20.52 (12.59, 28.24)	50.39 (43.62, 57.06)	45.49 (39.34, 52.21)	−0.31 (−0.42, -0.21)	86 (43, 167)	151 (72, 295)	75.40 (−25.53, 359.36)	0.25 (0.09, 0.54)	0.21 (0.07, 0.44)	−0.73 (−0.92, −0.54)
Mexico	15,092 (12,738, 18,088)	17,323 (15,042, 19,836)	14.78 (8.50, 21.28)	15.13 (12.93, 17.75)	14.60 (12.58, 16.93)	−0.61 (−0.79, −0.42)	625 (508, 739)	856 (698, 1,013)	36.87 (0.88, 80.00)	0.64 (0.52, 0.76)	0.68 (0.56, 0.80)	−0.05 (−0.71, 0.61)
Philippines	13,432 (11,575, 15,496)	19,299 (17,123, 21,722)	43.68 (38.44, 50.06)	18.92 (16.67, 21.44)	17.02 (15.18, 19.00)	−0.26 (−0.32, −0.20)	992 (532, 1,733)	1,284 (655, 1,929)	29.35 (−21.32, 155.79)	1.29 (0.74, 2.15)	1.20 (0.63, 1.79)	−0.02 (−0.18, 0.15)
Ethiopia	10,419 (9,020, 12,075)	18,744 (16,275, 21,611)	79.91 (72.77, 87.31)	19.30 (17.17, 21.77)	16.62 (14.91, 18.65)	−0.53 (−0.57, −0.49)	899 (333, 1,800)	1,150 (538, 2,286)	28.00 (−41.38, 223.83)	1.95 (0.76, 3.81)	1.24 (0.60, 2.41)	−1.48 (−1.58, −1.38)
Egypt	6,202 (5,416, 7,045)	9,743 (8,498, 11,150)	57.08 (46.10, 68.26)	9.86 (8.75, 11.06)	9.29 (8.25, 10.39)	−0.27 (−0.32, −0.21)	880 (605, 1,241)	798 (612, 1,171)	−9.23 (−37.92, 41.27)	1.48 (1.03, 1.99)	0.92 (0.65, 1.25)	−1.10 (−1.21, −0.99)
Vietnam	13,018 (11,492, 14,887)	17,292 (15,694, 18,870)	32.83 (23.91, 42.39)	17.60 (15.80, 19.62)	17.79 (16.01, 19.57)	−0.30 (−0.41, −0.20)	1,724 (790, 3,086)	1,578 (764, 3,027)	−8.42 (−62.32, 150.57)	2.33 (1.07, 4.16)	1.61 (0.78, 3.04)	−1.26 (−1.35, −1.17)
DR Congo	5,183 (4,346, 6,062)	11,430 (9,648, 13,678)	120.52 (108.00, 130.11)	12.59 (10.97, 14.32)	11.87 (10.34, 13.55)	−0.16 (−0.19, −0.13)	521 (216, 1,124)	1,004 (357, 2,137)	92.74 (−35.05, 370.99)	1.23 (0.54, 2.51)	1.20 (0.43, 2.54)	−0.15 (−0.27, −0.03)
Iran	4,498 (3,738, 5,443)	5,045 (4,335, 5,875)	12.15 (4.87, 21.73)	6.95 (5.96, 8.06)	6.43 (5.53, 7.45)	−0.12 (−0.18, −0.07)	239 (124, 411)	218 (123, 344)	−9.05 (−51.28, 120.47)	0.42 (0.22, 0.70)	0.29 (0.17, 0.47)	−0.94 (−1.30, −0.58)
Turkey	5,244 (4,419, 6,238)	6,308 (5,407, 7,332)	20.29 (13.15, 29.05)	8.84 (7.57, 10.20)	7.97 (6.78, 9.34)	−0.50 (−0.64, −0.36)	269 (119, 477)	244 (174, 340)	−9.28 (−50.61, 113.25)	0.51 (0.23, 0.93)	0.28 (0.20, 0.39)	−2.44 (−2.62, −2.25)
Thailand	7,419 (6,430, 8,502)	8,370 (7,545, 9,236)	12.82 (1.44, 24.15)	13.84 (12.21, 15.49)	12.41 (11.01, 14.01)	−0.54 (−0.62, −0.45)	568 (282, 991)	851 (536, 1,256)	49.74 (−18.94, 268.42)	1.12 (0.55, 1.95)	1.05 (0.70, 1.57)	−0.75 (−1.13, −0.36)
Tanzania	5,188 (4,542, 5,975)	11,222 (9,844, 12,905)	116.30 (105.18, 127.82)	17.92 (16.00, 20.28)	16.61 (14.93, 18.62)	−0.23 (−0.26, −0.21)	397 (180, 788)	417 (218, 746)	4.88 (−57.68, 151.33)	1.26 (0.61, 2.40)	0.71 (0.38, 1.26)	−1.89 (−1.98, −1.81)
South Africa	4,793 (4,039, 5,700)	6,700 (5,772, 7,815)	39.80 (34.83, 46.01)	12.45 (10.75, 14.43)	10.89 (9.41, 12.67)	−0.40 (−0.49, −0.30)	183 (112, 289)	288 (202, 407)	57.05 (−5.85, 169.36)	0.52 (0.32, 0.81)	0.47 (0.33, 0.67)	−0.70 (−1.02, −0.38)
Burma	4,687 (4,012, 5,455)	5,545 (4,883, 6,359)	18.31 (11.93, 25.79)	10.98 (9.57, 12.52)	10.01 (8.87, 11.45)	−0.23 (−0.28, −0.19)	479 (195, 940)	463 (258, 884)	−3.30 (−60.79, 184.38)	1.10 (0.44, 2.12)	0.86 (0.49, 1.65)	−1.04 (−1.24, −0.85)
Colombia	5,411 (4,545, 6,400)	6,815 (5,885, 7,791)	25.95 (17.82, 35.67)	15.17 (13.00, 17.62)	14.89 (12.72, 17.25)	−0.07 (−0.15, 0.01)	182 (156, 205)	312 (270, 363)	71.32 (38.70, 107.66)	0.56 (0.47, 0.63)	0.60 (0.52, 0.70)	0.65 (−0.20, 1.51)

Mortality and health loss showed more favorable long-term trends. The number of deaths decreased by 5.8%, from 74,378 to 70,042, and the ASMR fell from 1.96 to 1.13 per 100,000 person-years (EAPC = −1.97, 95% CI: −2.15 to −1.79) (Table 1). Total DALYs declined by 25.5% over the same period, with the ASDR decreasing from 119.33 to 64.94 per 100,000 (EAPC = −2.20, 95% CI: −2.38 to −2.03) (online suppl. Table 4). Joinpoint regression showed an overall decline in ASRs during 1990–2023, but incidence reached its lowest level around 2010 and increased thereafter, particularly after 2019 (Fig. 1; online suppl. Fig. 1).

**Fig. 1.**
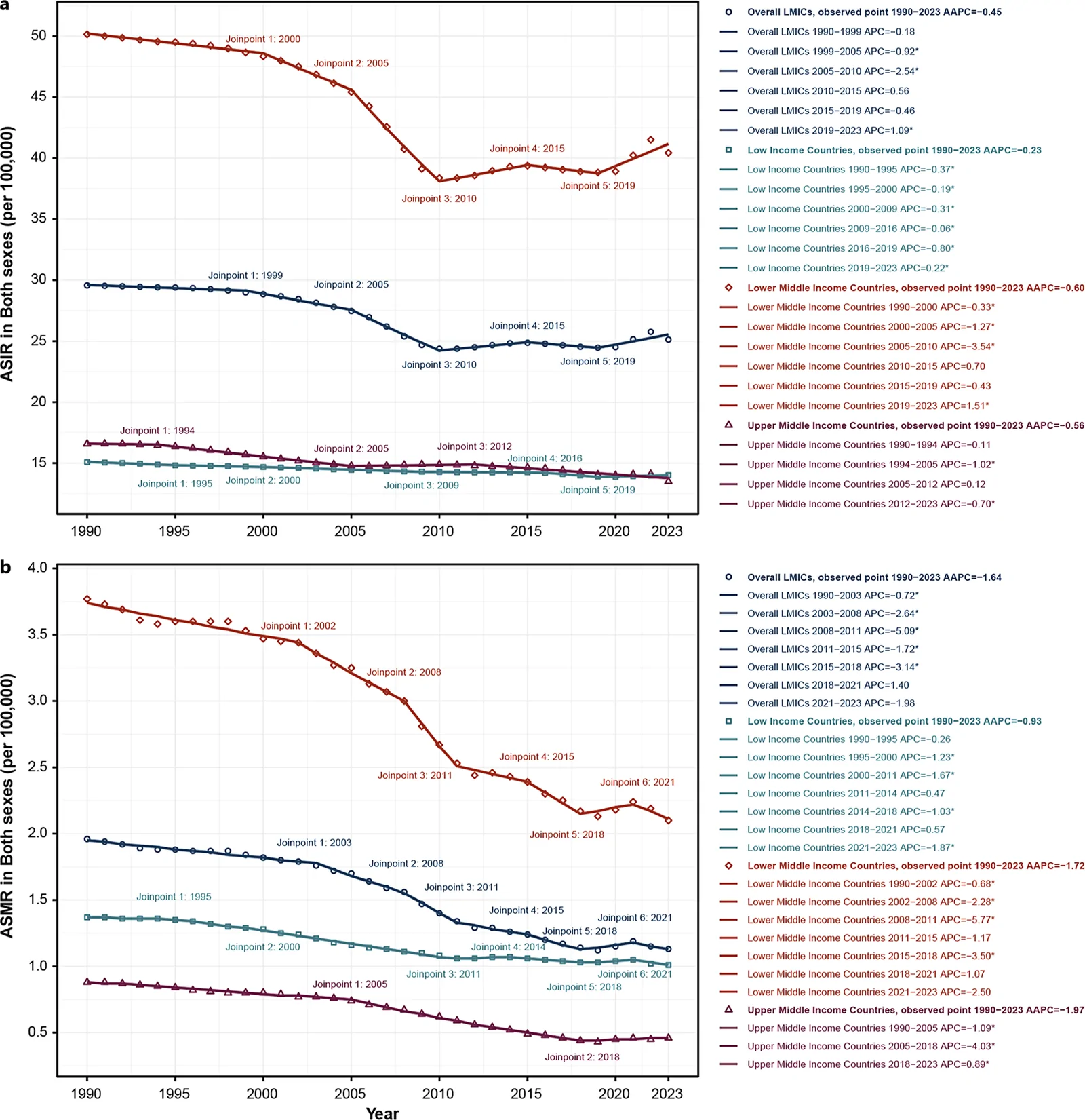
Joinpoint analysis of ASIR and ASMR of encephalitis in LMICs, 1990–2023. Shown are the observed trends and Joinpoint-segmented trends for ASIR (**a**) and ASMR (**b**) per 100,000 population in both sexes, overall, and by World Bank income group. APCs are presented for each segment, with asterisks (*) indicating statistical significance (*p* < 0.05). The AAPC over the entire study period (1990–2023) is also provided for each group.

### Geographic Variation across Regions and Countries

Encephalitis burden varied markedly across LMIC regions and countries. In 2023, the largest absolute burdens were concentrated in highly populous countries. India accounted for the highest number of incident cases (768.30 thousand), followed by China (199.73 thousand) (Fig. 2a). India also had the largest number of prevalent cases, deaths, and DALYs (Fig. 3a; online suppl. Fig. 2a, 3a; online suppl. Table 4).

**Fig. 2.**
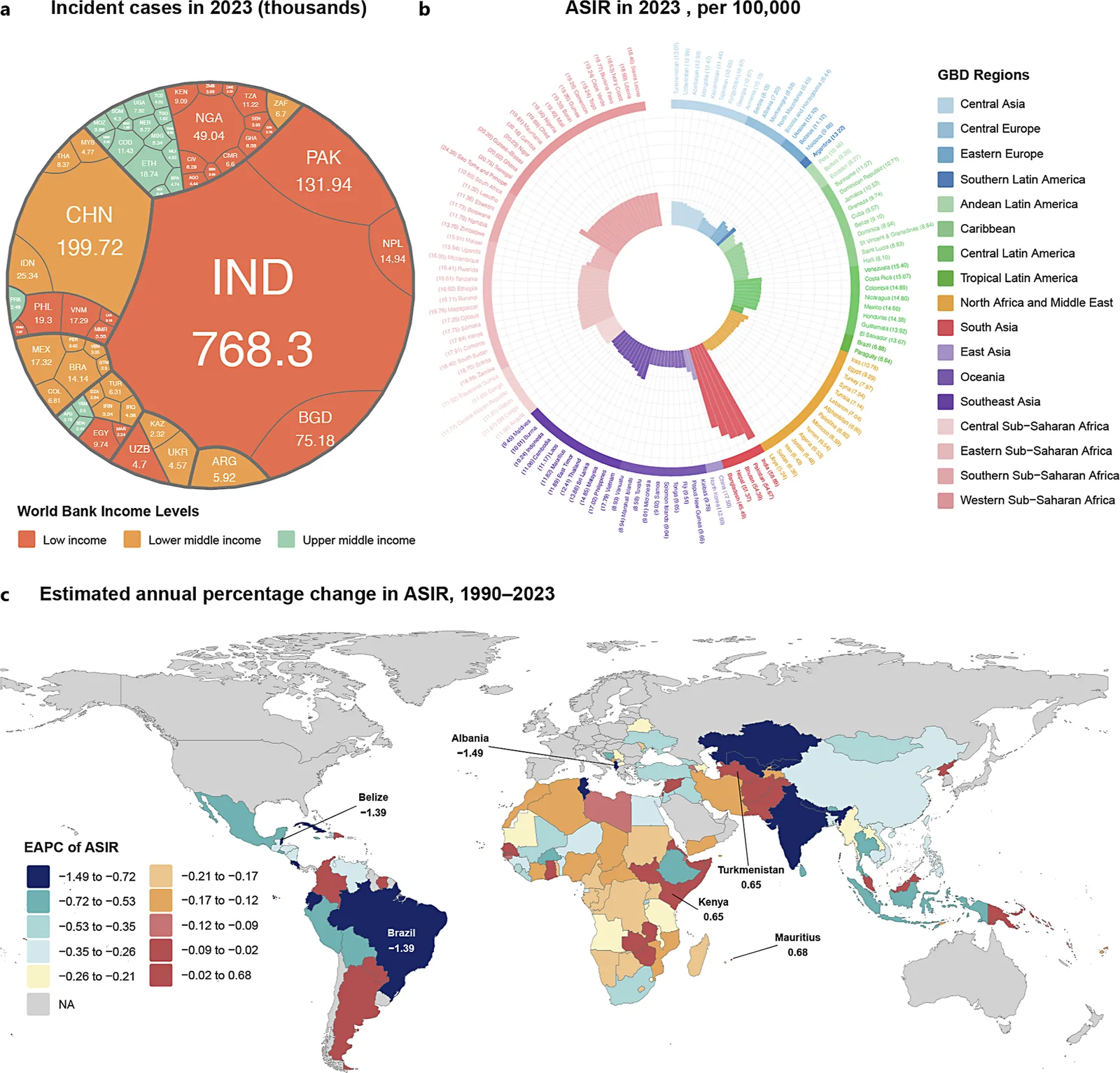
Incidence of encephalitis in LMICs in 2023 and temporal trends from 1990 to 2023. **a** Incident cases in LMICs in 2023 (thousands). **b** ASIR in LMICs in 2023, per 100,000 population. **c** EAPC in ASIR in LMICs from 1990 to 2023.

**Fig. 3.**
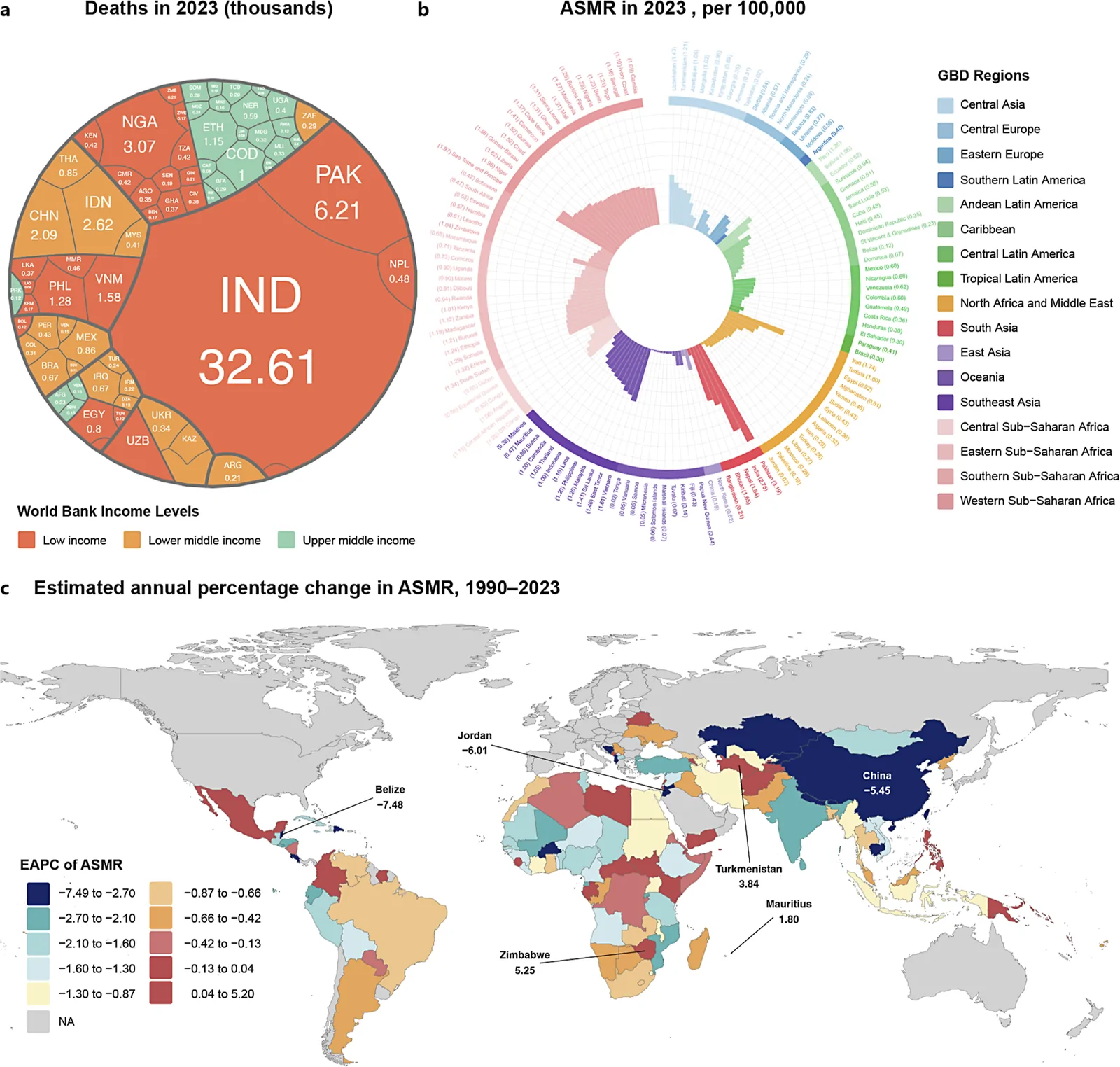
Mortality of encephalitis in LMICs in 2023 and temporal trends from 1990 to 2023. **a** Deaths in LMICs in 2023 (thousands). **b** ASMR in LMICs in 2023, per 100,000 population. **c** EAPC in ASMR in LMICs from 1990 to 2023.

At the regional level, South Asia had the highest ASIR among LMIC regions, whereas Western sub-Saharan Africa had the highest ASMR and ASDR (Fig. 2b, 3b; online suppl. Fig. 3b). Prevalence showed a broadly similar geographic distribution, with regional variation in ASPR shown in online supplementary Fig. 2b. Temporal trends also differed substantially between countries, with EAPCs showing both decreases and increases across LMICs (Fig. 2c, 3c; online suppl. Fig. 2c, 3c; online suppl. Tables 5–7).

### Age- and Sex-Specific Patterns of Encephalitis Burden

Age-specific analyses showed that encephalitis burden was concentrated at both ends of the life course. In 2023, age-specific incidence and mortality rates were highest in children and adolescents, then rose again among older adults aged 70 years and above in both sexes (Fig. 4). Age-specific DALY rates peaked in children younger than 10 years, declined through adulthood, and increased modestly again in later life (online suppl. Fig. 4).

**Fig. 4.**
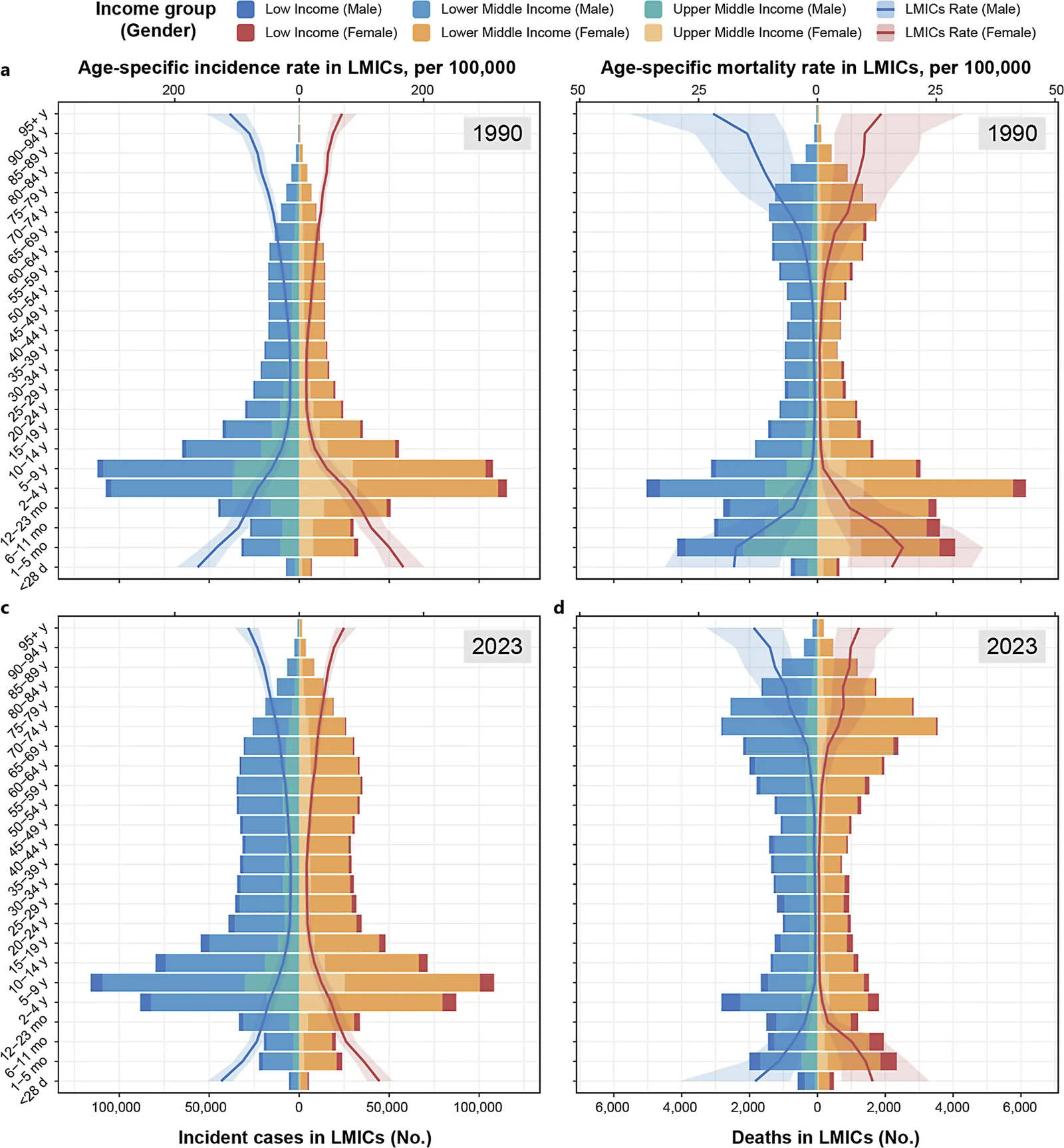
Age- and sex-specific burden of encephalitis in LMICs in 1990 and 2023. **a** Age-specific incidence rates (per 100,000) and incident cases in LMICs by sex in 1990. **b** Age-specific mortality rates (per 100,000) and deaths in LMICs by sex in 1990. **c** Age-specific incidence rates (per 100,000) and incident cases in LMICs by sex in 2023. **d** Age-specific mortality rates (per 100,000) and deaths in LMICs by sex in 2023. Bars represent the number of incident cases or deaths in LMICs, and lines represent age-specific rates. Age-groups include <28 days, 1–5 months, 6–11 months, 12–23 months, 2–4 years, 5–9 years, followed by 5-year age intervals up to ≥95 years.

Across nearly all age-groups, males carried a slightly higher burden than females. In 2023, the ASIR was 25.83 per 100,000 in males and 24.51 per 100,000 in females (Table 1). Similar patterns were observed for ASMR and ASDR. Over time, declines in ASMR and ASDR were somewhat greater among females than among males (Table 1; Fig. 4; online suppl. Fig. 4).

### Age-Period-Cohort Effects

Age-period-cohort analyses showed distinct age, period, and cohort patterns across burden indicators (Fig. 5). Net drift was negative for incidence, prevalence, deaths, and DALYs across income groups, and local drift was predominantly negative across age-groups. In general, reductions were more pronounced at younger and middle ages, whereas slower declines or modest upward deviations were more likely to appear in older age-groups, particularly for deaths and DALYs (Fig. 5a).

**Fig. 5.**
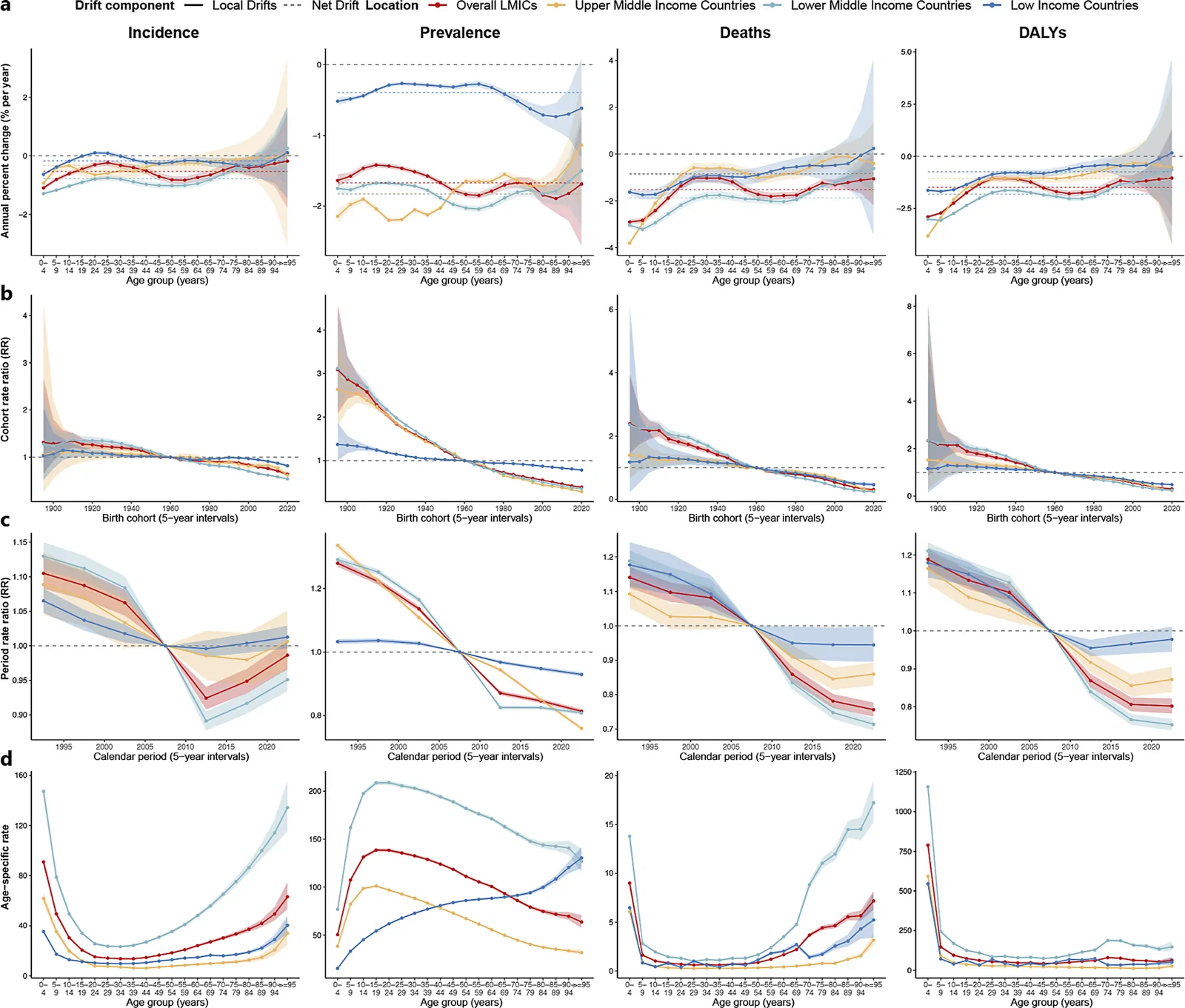
Age-period-cohort analysis of encephalitis burden indicators (incidence, prevalence, deaths, and DALYs) in LMICs, 1990–2023. **a** Net drift and local drift. **b** Cohort effects. **c** Period effects. **d** Longitudinal age curves. Solid lines and shaded areas represent the estimated values and 95% CIs, respectively. Results are stratified by income group.

Cohort effects showed progressive improvement across successive birth cohorts, with CRRs declining from earlier to more recent cohorts (Fig. 5b). Period effects differed by outcome: incidence declined until around 2010–2015 and then increased, whereas deaths and DALYs declined more steadily over time (Fig. 5c). Longitudinal age curves confirmed a U-shaped incidence pattern, with higher fitted rates in early life and older adulthood; deaths and DALYs were also concentrated at the extremes of age (Fig. 5d). Sex-specific APC results are presented in online supplementary Figures 5 and 6.

### Sociodemographic Development and Income-Group Differences

Encephalitis burden was inversely associated with sociodemographic development. Using pooled country-year observations in 2023 across the 131 LMICs, ASIR, ASMR, and ASDR were all negatively correlated with SDI (all *p* < 0.001), with the strongest correlation observed for ASIR (ρ = −0.59), followed by ASDR (ρ = −0.56) and ASMR (ρ = −0.41) (Fig. 6; online suppl. Fig. 7).

**Fig. 6.**
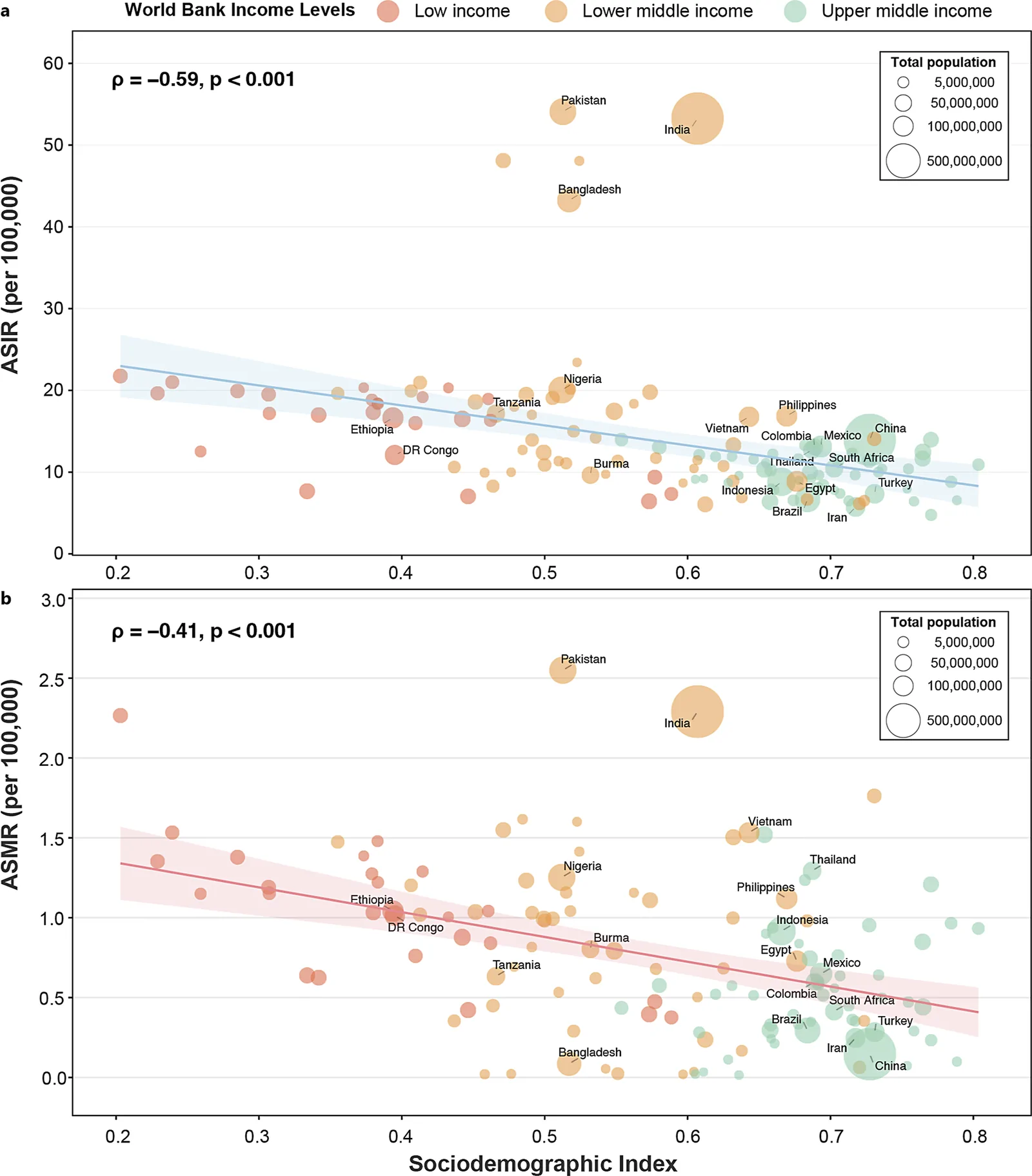
Association between encephalitis burden and SDI in LMICs. **a** Relationship between ASIR of encephalitis and the SDI. **b** Relationship between ASMR of encephalitis and SDI. Each point represents a country in 2023 with point size proportional to total population. The solid line represents the regression trend, while the shaded area indicates the 95% CIs.

A similar gradient was seen across World Bank income groups. In 2023, lower-middle-income countries (LMCs) bore the highest burden, including the highest ASIR, ASMR, and ASDR (Table 1; online suppl. Table 4). Despite this high burden, LMCs showed the fastest decline in ASIR, whereas the steepest reductions in ASMR and ASDR occurred in upper-middle-income countries (Table 1; online suppl. Table 4).

### Decomposition of Changes in Absolute Burden

Decomposition analysis identified different drivers of changes in burden between 1990 and 2023. Increases in incident and prevalent cases were driven mainly by population growth, whereas reductions in deaths and DALYs were driven mainly by decreases in age-specific rates (Fig. 7; online suppl. Fig. 8).

**Fig. 7.**
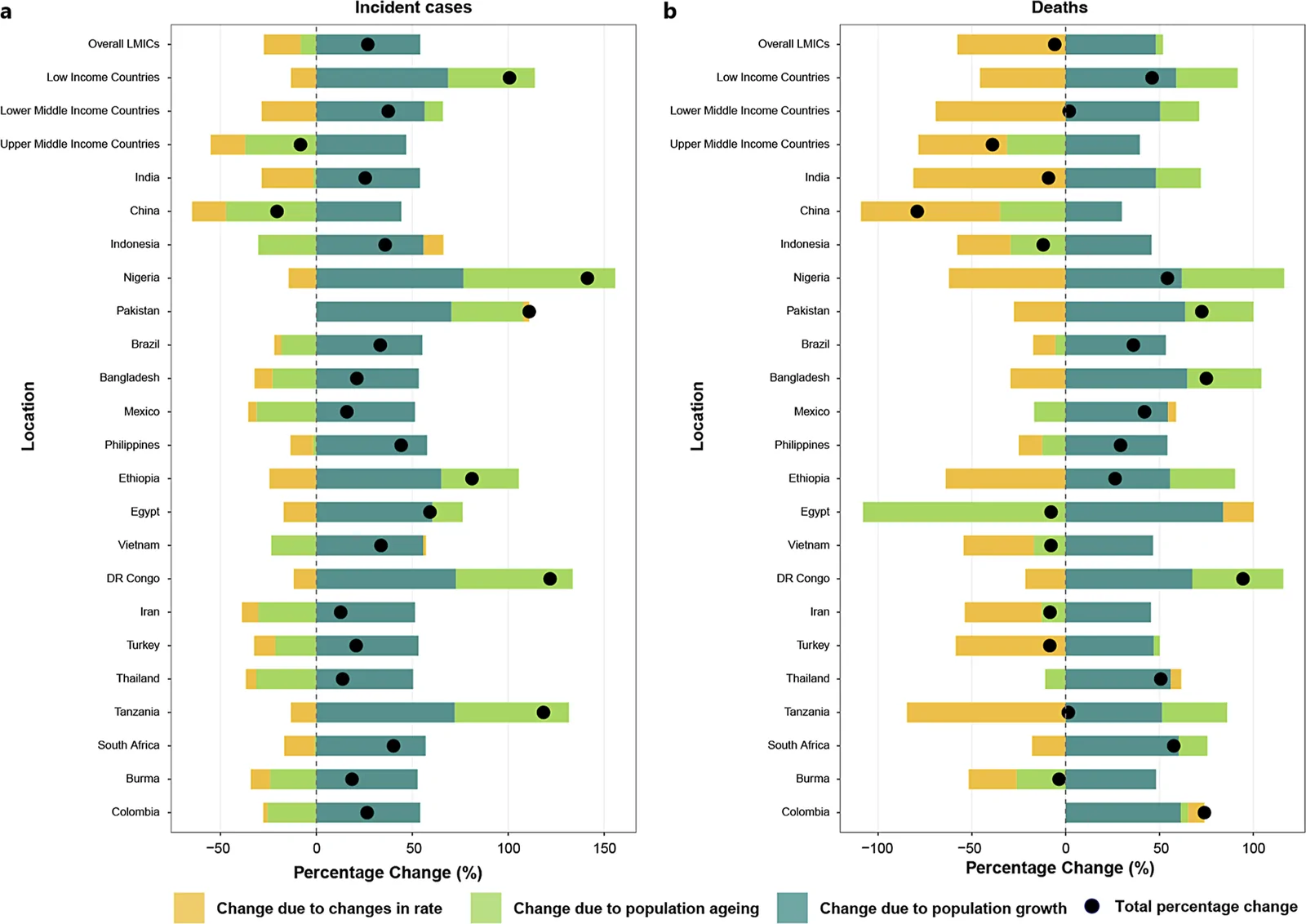
Decomposition of changes in encephalitis burden in LMICs, 1990–2023. **a** The percentage change in incident cases. **b** The percentage change in deaths attributed to changes in age-specific rates, population aging, and population growth, along with the total percentage change.

## Discussion

Although the age-standardized burden of encephalitis declined across LMICs between 1990 and 2023, the disease continued to impose a substantial absolute burden, with incident and prevalent cases still rising over time. This contrast suggests that demographic expansion has offset part of the progress reflected in rate-based measures. In addition, incidence and prevalence showed an upward shift after approximately 2010, which became more evident after 2019. The uneven patterns observed across age-groups, regions, income strata, and sociodemographic levels further suggest that improvements in prevention, diagnosis, and care have not been shared equally across countries.

The present study also highlights why an LMIC-focused analysis is needed beyond prior global assessments. Global averages are useful for describing broad trends, but they may obscure substantial heterogeneity within LMICs, where encephalitis burden is shaped not only by pathogen exposure but also by differences in vaccine coverage, surveillance capacity, etiologic diagnosis, and access to acute care. These differences also influence case ascertainment and outcome reporting because access to cerebrospinal fluid analysis, pathogen-specific testing, neuroimaging, specialist care, and vital registration remains uneven across many LMICs. By examining development gradients, decomposition patterns, and age-period-cohort effects specifically within LMICs, this analysis provides information that is less visible in broader global summaries. This distinction is particularly important for infection-focused public health planning because the determinants of encephalitis burden in resource-limited settings are likely to be more closely tied to vaccine-preventable infections, arboviral transmission, diagnostic access, and health-system constraints than is apparent from global estimates alone.

An important feature of the present analysis is the coexistence of declining ASRs and a persistently high absolute burden. Decomposition analysis showed that increases in incident and prevalent cases were driven mainly by population growth, whereas reductions in deaths and DALYs were largely attributable to declines in age-specific rates. This pattern suggests that demographic expansion continues to sustain a large number of encephalitis cases even as individual-level risk and severe outcomes have improved. In practical terms, this means that reductions in age-standardized burden do not necessarily translate into lower service demand, especially in populous LMICs. Health systems may therefore face continuing pressure from encephalitis even when population-level rates appear to improve. This has direct implications for planning because prevention alone is unlikely to be sufficient; continued investment is also needed in diagnostic capacity, referral pathways, inpatient management, and rehabilitation services.

The temporal shift observed after approximately 2010, together with the more noticeable upward change after 2019 for incidence and, to a lesser extent, prevalence, warrants cautious interpretation. Because encephalitis represents a heterogeneous syndrome, these trends likely reflect the combined behavior of infectious, post-infectious, and immune-mediated conditions rather than changes in a single uniform disease entity. Similar reversals have been noted in previous global analyses, suggesting that this pattern is unlikely to be entirely incidental. Several non-mutually exclusive mechanisms may be relevant. Improved surveillance and greater clinical recognition may have increased case ascertainment in some settings. Climate-sensitive changes in vector ecology and geographic suitability may also have altered the transmission risk of arboviral encephalitides, including JE and West Nile virus [20–23]. Public health disruption is another plausible contributor, particularly where outbreaks, fragile health infrastructure, or interruptions in routine immunization weakened protection against vaccine-preventable causes of encephalitis [24]. The more recent upward shift after 2019 may also be related to direct or indirect effects of the COVID-19 pandemic, such as SARS-CoV-2-associated encephalitis, delayed care seeking, disrupted vaccination services, reduced access to specialist care, or changes in surveillance and reporting practices [25–28]. However, these explanations should be regarded as plausible hypotheses rather than causal conclusions. Long-term trends may also have been influenced by evolving diagnostic practice, including improved recognition of immune-mediated encephalitides through antibody testing and by changing susceptibility to central nervous system infections in populations receiving immunomodulatory or immunosuppressive therapies. These factors cannot be directly evaluated within the aggregate GBD encephalitis category, but they should be considered when interpreting temporal and regional differences.

Age patterns in this study were consistent with established biological vulnerability at both ends of the life course. The age-period-cohort results showed that incidence followed a U-shaped age pattern, with relatively high fitted rates in early life and a renewed increase in older age, while deaths and DALYs were concentrated in early childhood and older adulthood. These findings are biologically plausible and align with immune immaturity in children, as well as immunosenescence, multimorbidity, and reduced physiological reserve in older adults [29–31]. Pediatric vaccination, maternal-child health services, and timely recognition of central nervous system infections in children remain essential in LMICs. At the same time, population aging in many LMICs may increase the share of severe encephalitis outcomes occurring in older adults, highlighting the need for rapid referral, neurocritical care access, and post-acute rehabilitation. Sex differences were present but modest. Males generally showed a slightly higher burden across several indicators, which is broadly consistent with previous epidemiological observations in neuroinfectious disease, although the supplementary age-period-cohort analyses suggested that recent period effects may not be identical between men and women [32–34].

Geographic and developmental inequalities were another prominent finding. South Asia had the highest incidence burden, whereas Western sub-Saharan Africa bore the heaviest mortality and DALY burden. LMCs carried the highest overall burden in 2023, and higher SDI was consistently associated with lower age-standardized burden. These patterns support the interpretation that encephalitis burden in LMICs is shaped not only by pathogen exposure but also by inequities in vaccination coverage, etiologic diagnosis, surveillance systems, and access to acute care. In high-incidence settings, endemic viral transmission, population density, and incomplete vaccine uptake may continue to sustain case numbers [6, 35]. In high-mortality settings, delayed diagnosis, restricted cerebrospinal fluid testing, limited antiviral availability, and weak neurocritical care capacity may contribute more directly to fatality and disability [6, 8, 9]. The inverse association with SDI further suggests that encephalitis burden functions, in part, as a marker of broader health-system capacity and public health preparedness in resource-limited settings [6, 8].

This study draws on the latest GBD 2023 estimates to provide a long-term and internally comparable assessment of encephalitis burden across 131 LMICs, a group often obscured when interpretation is dominated by global averages. By combining EAPC, Joinpoint regression, age-period-cohort modeling, decomposition analysis, and SDI correlation, it offers a more integrated view of temporal change, population structure, and development-related inequality, with direct relevance to surveillance, diagnostic prioritization, and health-system planning in resource-limited settings.

Several limitations should be acknowledged. First, GBD estimates are model based and may not fully match observed values, particularly in LMICs where surveillance systems, diagnostic capacity, healthcare utilization, and mortality reporting vary substantially. Restricted access to cerebrospinal fluid testing, neuroimaging, pathogen-specific diagnostics, specialist care, and reliable death certification may lead to under-recognition or misclassification. Second, because the analysis relied on the aggregate GBD encephalitis category, it was not possible to distinguish clinically important subgroups such as vaccine-preventable encephalitis, herpes simplex virus-related encephalitis, arboviral encephalitis, post-infectious encephalitis, or autoimmune encephalitis. Accordingly, temporal and geographical patterns should be interpreted as the combined signal of multiple conditions with different prevention and treatment pathways. Third, potential explanations for recent changes, including climate-sensitive vector ecology, outbreaks, healthcare disruption, and COVID-19-related effects, should be regarded as hypotheses rather than confirmed drivers. Finally, LMICs were defined using the current World Bank income classification as a static grouping framework, which improved comparability but did not capture changes in income category over time. Consequently, some countries may have experienced changes in economic status during the study period that were not captured by this classification approach.

## Conclusion

Encephalitis continues to impose a substantial burden in LMICs. Although ASIR, ASMR, and ASDR declined from 1990 to 2023, absolute incident and prevalent cases remained high, and incidence showed a recent upward shift after a prolonged decline. Persistent disparities across age, region, income group, and sociodemographic development indicate that LMIC-specific challenges are not fully captured by global averages. Strengthening prevention, surveillance, diagnostic capacity, and acute-care preparedness will be essential to reduce encephalitis-related morbidity and mortality in resource-limited settings.

## Acknowledgments

We acknowledge the GBD 2023 study collaborators for providing open-access data used in this research.

## Statement of Ethics

This study was based exclusively on publicly available, de-identified data from the Global Burden of Disease (GBD) 2023 study. Ethical approval and informed consent were not required.

## Conflict of Interest Statement

The authors declare that they have no competing personal interests or relationships that could be perceived to influence the work reported in this manuscript.

## Funding Sources

This work was supported by the Science and Technology Innovation 2030 Major Projects of China (Grant No. 2021ZD0204300) and the Beijing Natural Science Foundation (Grant No. L256041).

## Author Contributions

Kun Fang: writing – original draft, writing – review and editing, data collection, and conceptualization. Huawei Zhang: writing – original draft, writing – review and editing, data collection, visualization, and conceptualization. Jinming Han and Chengliang Yin: writing – review and editing and supervision. Jiahao Song, Xiangjun He, and Xingxu Ji: writing – review and editing and data collection. Changqing Liu and Daiquan Gao: writing – review and editing, supervision, and funding acquisition.

## Data Availability

All data analyzed in this study were obtained from the GBD 2023 study and are publicly available through the GBD Results Tool provided by the Institute for Health Metrics and Evaluation (IHME) at https://vizhub.healthdata.org/gbd-results/.
